# The OX40/OX40L Axis Regulates T Follicular Helper Cell Differentiation: Implications for Autoimmune Diseases

**DOI:** 10.3389/fimmu.2021.670637

**Published:** 2021-06-21

**Authors:** NanNan Fu, Fang Xie, ZhongWen Sun, Qin Wang

**Affiliations:** ^1^ School of Biology & Basic Medical Sciences, Medical College of Soochow University, Suzhou, China; ^2^ Department of Medical Technology, Suzhou Vocational Health College, Suzhou, China

**Keywords:** OX40, OX40L, Tfh cells, differentiation, autoimmune diseases

## Abstract

T Follicular helper (Tfh) cells, a unique subset of CD4^+^ T cells, play an essential role in B cell development and the formation of germinal centers (GCs). Tfh differentiation depends on various factors including cytokines, transcription factors and multiple costimulatory molecules. Given that OX40 signaling is critical for costimulating T cell activation and function, its roles in regulating Tfh cells have attracted widespread attention. Recent data have shown that OX40/OX40L signaling can not only promote Tfh cell differentiation and maintain cell survival, but also enhance the helper function of Tfh for B cells. Moreover, upregulated OX40 signaling is related to abnormal Tfh activity that causes autoimmune diseases. This review describes the roles of OX40/OX40L in Tfh biology, including the mechanisms by which OX40 signaling regulates Tfh cell differentiation and functions, and their close relationship with autoimmune diseases.

## Introduction

Many autoimmune diseases such as systemic lupus erythematosus (SLE) and rheumatoid arthritis (RA) are characterized by autoantibody production. A specialized cell subset named T follicular helper (Tfh) cells has attract much attention because of their requirement for B cell help and the production of high affinity class-switched antibodies. Tfh cells, located in lymphoid follicles, belong to a distinct CD4^+^ T subset. They are essential for generation of effective and long-lived humoral immune responses. Several pairs of costimulatory molecules have been demonstrated to control Tfh development and function. OX40/OX40L is one of them. OX40 and OX40L play a critical role in enhancement of immune responses and participate in the development of autoimmune diseases. Recently, it was reported that the OX40/OX40L interaction is required for the functions of Tfh cells. This article focuses on the effects of OX40/OX40L signaling on Tfh cells and their roles in the pathogenesis of autoimmune diseases.

## Differentiation and Functions of Tfh Cells

In 2000, Schaerli and Breifeld found that there is a subset of CD4^+^ T cells in lymphoid follicles, called Tfh cells, which express CXCR5, ICOS and CD40L (1). They are obviously distinct from other Th cells in two aspects. First, while CXCR5 is only expressed temporarily on other Th cell subpopulations when they are activated, while its expression on Tfh cells persists for a long time. Upregulated CXCR5 and downregulated CCR7 facilitate the migration of Tfh cells from the T cell area to CXCL13-rich B lymphoid follicles where they interact with B cells. Second, unlike Th1, Th2 and Th17 cells, the differentiation of Tfh cells proceeds through multiple stages, including initiation, maintenance and full polarization. A variety of cytokines, transcription factors and surface molecules are involved in these process (2–4).

ICOS, PD-1, Bcl-6, BTLA, CD40L, IL-21, IL-6R, SAP and IL-21R are shown to be highly expressed in mouse and human Tfh cells, indicating that these molecules may play critical roles in promoting the development and maintenance of Tfh cells and regulating their functions (4–7). Bcl-6 is recognized as an essential transcription factor for regulation of Tfh cell differentiation. Bcl6 controls Tfh differentiation by antagonizing Blimp-1 and other transcription factors which are also important for Th1, Th2 and Th17 cells. GCs are absent in Bcl-6-deficient mice since Bcl-6 deficient CD4^+^ T cells do not support the GC reaction. The expression of Bcl-6 in Tfh cells is mainly driven by IL-6, IL-21 and certain downstream transcription factors including STAT1 and STAT3 (8, 9). Recently, a feed-forward loop mediated by the transcription factors Bcl-6 and Tox2 is reported to promote the Tfh program (10). Bcl-6 upregulates Tox2 expression, which further drives Bcl-6 expression and Tfh development. ICOS/ICOSL molecules are involved in every stage of Tfh cell differentiation. Mathieu et al. transferred ICOS-Y181F into mice to block ICOS-mediated PI3K activation and found that the number of Tfh cells in the spleen was significantly reduced. At the same time, ICOS-PI3K was also found to be essential for upregulating IL-21 and IL-4, which are key factors for Tfh function (4, 11). These multiple factors drive Tfh differentiation. The first stage of Tfh differentiation is initiated by interaction with a professional antigen-presenting cell (APC), such as a dendritic cell (DC). After T cell priming, Bcl-6 and CXCR5 expression is upregulated on CD4^+^ T cells to facilitate Tfh cell migration to the T-B border. Then, the second stage begins. This is a B cell-dependent phase of Tfh differentiation that is regulated by ICOS/ICOSL signaling. In the third stage, Tfh cells and B cells migrate to GCs, where B cells continuously help Tfh cells to promote their full polarization. GC Tfh cells are in a further polarized Tfh cell state, with elevated expression of Bcl-6, CXCR5, PD-1 and ICOS.

The most important function of Tfh cells is to provide help to B cells (12). They are necessary for GC formation, high-affinity B cell selection, and generation of memory B cells and plasma cells (13, 14). At the T-B border, B cells present antigens to Tfh cells and only those cells presenting antigens with high affinity obtain Tfh help and then enter GCs (15). Most B cell responses cannot progress without the help of Tfh cells. GCs are recognized as the essential sites of B cell mutation and antibody affinity maturation. After GC Tfh cells recognize the antigens presented by GC B cells in the follicle light zone, they provide signals for GC B cell proliferation and migration to the dark zone, where B cells will undergo somatic hypermutation (16). Moreover, GC Tfh cells promote the development of long-term humoral immunity by generating memory B cells and high-affinity plasma cells. The effect of Tfh cells on B cell differentiation within GCs and extrafollicular areas depends on numerous signals, such as IL-21 and CD40 signals. IL-21 is the most potent cytokine driving plasma cell differentiation in both mice and humans (17–21). IL-21 induces both Bcl-6 and Blimp-1 expression in B cells, in which Bcl-6 promotes GC B cell proliferation and Blimp-1 is critical for plasma cell differentiation (17, 22, 23). IL-21 signaling is dependent on the activation of STAT3 and STAT5 (24). CD40L/CD40 engagement is central to the maintenance of GC B cells. Provision of CD40L protein *in vitro* was found to inhibit apoptosis of GC B cells (25–27). Schirock et al. reported that a critical ECM:α_v_ integrin axis specifically regulated prolonged Tfh positioning within the GCs and supported the generation of long-lived plasma cells but not memory B cells (28).

## OX40 and OX40L Molecules

### Structure and Expression of OX40 and OX40L

OX40 (also called ACT35, CD134 or TNFRSF4), belonging to the TNFR superfamily, is a type 1 transmembrane protein containing 249 amino acids with a 49 amino acids in cytoplasmic tail and a 186 amino acids in extracellular region (29, 30). OX40 protein was first recognized on activated rat CD4^+^ T cells in 1987. Subsequently, OX40 expression was also found expressed in mice and humans (31–33). OX40 is mainly expressed on activated CD4^+^ T cells and CD8^+^ T cells, whereas its expression level is relatively low on NK cells and NKT cells (29, 34–37). OX40L (also named as CD252, TNFSF4, CD134L or gp34), the ligand of OX40, is a type II glycoprotein with a 23 amino acids cytoplasmic tail and a 133 amino acids extracellular domain (38). As a member of the TNF superfamily, it is expressed as a trimer. OX40L was initially identified as gp34 protein on human T-cell leukemia virus transformed cells in 1985 (39). Later, it was found that OX40L is mainly expressed on antigen presenting cells, such as B cells and dendritic cells (40–42). Similar to other members of the TNF family, the OX40 signal transduces through TNF receptor related factors (TRAFs). OX40 signal is transduced to T cells *via* TRAF2 and TRAF5 *in vivo* and TRAF1, TRAF3 and TRAF5 *in vitro* (43–47).

The expression of OX40 and OX40L is regulated by many factors. OX40 expression is induced on T cells by TCR, CD28/CD80, CD40/CD40L and other signals and peaks at 48-72 hours following T cell activation (34, 48–50). TCR signaling can initiate the expression of OX40 on a variety of cells, while CD28 and other cytokines can further promote its expression on activated T cells (32). It has also been reported that IL-2, IL-4 and TNF can enhance or prolong OX40 expression. Sun et al. found that IL-2, TNF-α and IFN-γ were highly expressed in liver tissues of animal models of nonalcoholic steatohepatitis, but only exogenous IL-2 stimulation could upregulate OX40 expression on CD4^+^ T cells (51). CD40 signaling and inflammatory signals transmitted by Toll-like receptors induce OX40L expression on antigen-presenting cells (41). Factors such as IL-18, IFN-γ, thymic stromal lymphopoietin (TSLP) and prostaglandin E2 can also promote the expression of OX40L (49, 52–55). In an inflammatory environment, upregulated OX40L expression on APCs ensures that activated OX40^+^ T cells receive OX40 signals from nearby cells (33).

### Functions of OX40 and OX40L

As a pair of costimulatory molecules, OX40/OX40L is required for T cell activation especially in the later phase of the immune response. OX40/OX40L plays critical roles in enhancing the function of effector T cells, maintaining their survival and inhibiting their apoptosis. Rogers et al. detected a significantly decreased percentage of antigen-specific T cells in OX40-deficient mice. Moreover, antiapoptotic molecules, such as Bcl-xL and Bcl-2, were obviously downregulated in OX40^-/-^ T cells and CD28^-/-^ T cells after antigen stimulation. When CD28^-/-^ T cells were stimulated with an anti-OX40 agonist antibody, the expression of Bcl-xL and Bcl-2 was increased, and T cell apoptosis was inhibited (56). OX40-deficient T cells normally proliferated and differentiated into effector T cells 2-3 days after activation of TCR signaling. However, the survival rate was significantly reduced after 12-13 days of activation, which indicated that OX40 signaling might not be essential for the early stage of T cell activation but might promote the proliferation of T cells and maintain their survival in the later stage (57). OX40/OX40L signaling is critical for differentiation of various Th cells. This signaling preferentially induces differentiation of naive CD4^+^ T cells into Th2 cells but promotes Th1 differentiation under the influence of antigens or IL-12 (58). OX40 was also reported to play important roles in differentiation of Th9 cells through the nonclassical NF–κB pathway by activating tumor necrosis factor receptor-associated factor 6 (TRAF6) (59). Which type of Th cell differentiation is promoted by this signal may be dependent on the environment it is involved. OX40 expression is usually downregulated after the effect phase of the primary T cell response and can be rapidly upregulated on memory T cells after secondary challenge with the same antigen again to subsequently activate and recruit memory effector T cells, suggesting that the OX40/OX40L interaction is required in the recall response (59). OX40 is also constitutively expressed on mouse Treg cells (60). Evidence has shown that OX40 signaling is essential for inhibiting Treg cell function. Jaquemin et al. reported that engagement of the OX40/OX40L axis resulted in Foxp3 downregulation in Tregs and decreased Treg-mediated suppression of effector T cell proliferation(OX40 upregulates BATF3 and BATF, which produce a closed chromatin configuration to repress Foxp3 expression in a Sirt1/7-dependent manner (61). However, there are also reports showing that OX40 agonists can enhance Treg cell proliferation and inhibit function. Gavin MA et al. found that the number of Treg cells in the spleen of OX40-deficient mice decreased, while the number of Treg cells in the thymus of OX40L-overexpressing mice increased, indicating that abnormal OX40/OX40L signaling interfered with the development of Treg cells (62).

In addition to its critical role in T cells, OX40/OX40L signaling can also promote the differentiation and maturation of DCs. Human immature DCs have no OX40L expression, whereas the expression of OX40L is rapidly induced after sCD40L stimulation. Ligation of OX40L upregulated the expression of CD80, CD86, CD54 and CD40 on mononuclear-derived DCs in the reversible phase, and could enhance the secretion of IL-4, IL-6, IL-12, TNF-α and IL-1β by 4- to 35- fold (41). This result indicates that the OX40L reverse signal enhanced the maturation of DCs. B cells are also an OX40L-expressing antigen-presenting cells that play an important role in formation of the GC (63). Cross-linking of OX40L on stimulated B cells significantly enhanced proliferative response and promoted immunoglobulin secretion (64). Morimoto et al. found CD134L engagement on human B cells increased IgG production rate per cell rather than increasing the number of plasma cells (65). Therefore, the OX40/OX40L bidirectional signal not only acts on T cells but also plays critical roles in differentiation and maturation of APCs, especially DCs and B cells (**Table 1**).

**Table 1:** OX40/OX40L functions in different cell types.

Cell types	OX40 or OX40L expression	Functions
T cells	OX40 and OX40L	Promotion of T cell activation and proliferation (57)
		Inhibition of T cell apoptosis (56)
		Enhancement of recall response (59)
		Promotion of Th differentiation (58, 59)
Tfh cells	OX40	Promotion of Tfh differentiation and maintenance
		Enhancement of Tfh function of helping B cells
Tregs	OX40	Inhibition of Treg function (60, 61)
		Promotion of Treg proliferation (62)
DCs	OX40L	Promotion of the differentiation and maturation of DCs (41)
B cells	OX40L	Promotion of B cell proliferation and Ig secretion (64, 65)
		Promotion of GC formation (63)

## OX40/OX40L Signaling in Tfh cells

### Expression of OX40 On Tfh Cells

OX40 is transiently expressed on CD4^+^ T cells after 12-24 hour of activation. As a distinct CD4^+^ T cell subset, Tfh cells in both mice and humans have been confirmed to express OX40. Adam L et al. found that OX40 and ICOS were coexpressed on peripheral blood Tfh cells of patients with primary biliary cholangitis (PBC) and primary sclerosing cholangitis (PSC). Compared with PBC patients, PSC patients had significantly upregulated OX40 and ICOS expression (66). Analysis of patients with rheumatoid arthritis (RA) showed an abundance of OX40-overexpressing Tfh cells, especially Tfh17 cells (67). Jiang et al. also reported OX40 expression on Tfh cells in a mouse model of myelodysplastic syndrome (MDS) (68). Tahiliani et al. found that in mice infected with vaccinia virus, OX40 was already expressed on pre-Tfh cells, and the expression level gradually increased with maturation of the Tfh cells (69). Therefore, OX40 is expressed during differentiation of Tfh cells and may play a role in Tfh development and functions.

### Differentiation and Maintenance of Tfh Cells

Many cytokines and costimulatory signals, such as IL-12, IL-6 and ICOS/GL50, have been reported as the key factors in differentiation of Tfh cells. Recently, OX40/OX40L is characterized as another important costimulatory signal to promote Tfh differentiation. It has been reported that Rouqin regulates Tfh cell differentiation by inhibiting the expression of ICOS and OX40 mRNA, suggesting a close correlation between OX40 and Tfh cells (70). Defects in Rc3h1 and Rc3h2 in T cells elevate the expression of OX40 and Irf4, leading to activation of the NF-κB pathway. Tfh cells and GC-B cells spontaneously differentiate in the absence of immunization (70). Therefore, OX40 may promote the differentiation of Tfh cells.

**Figure 1:**
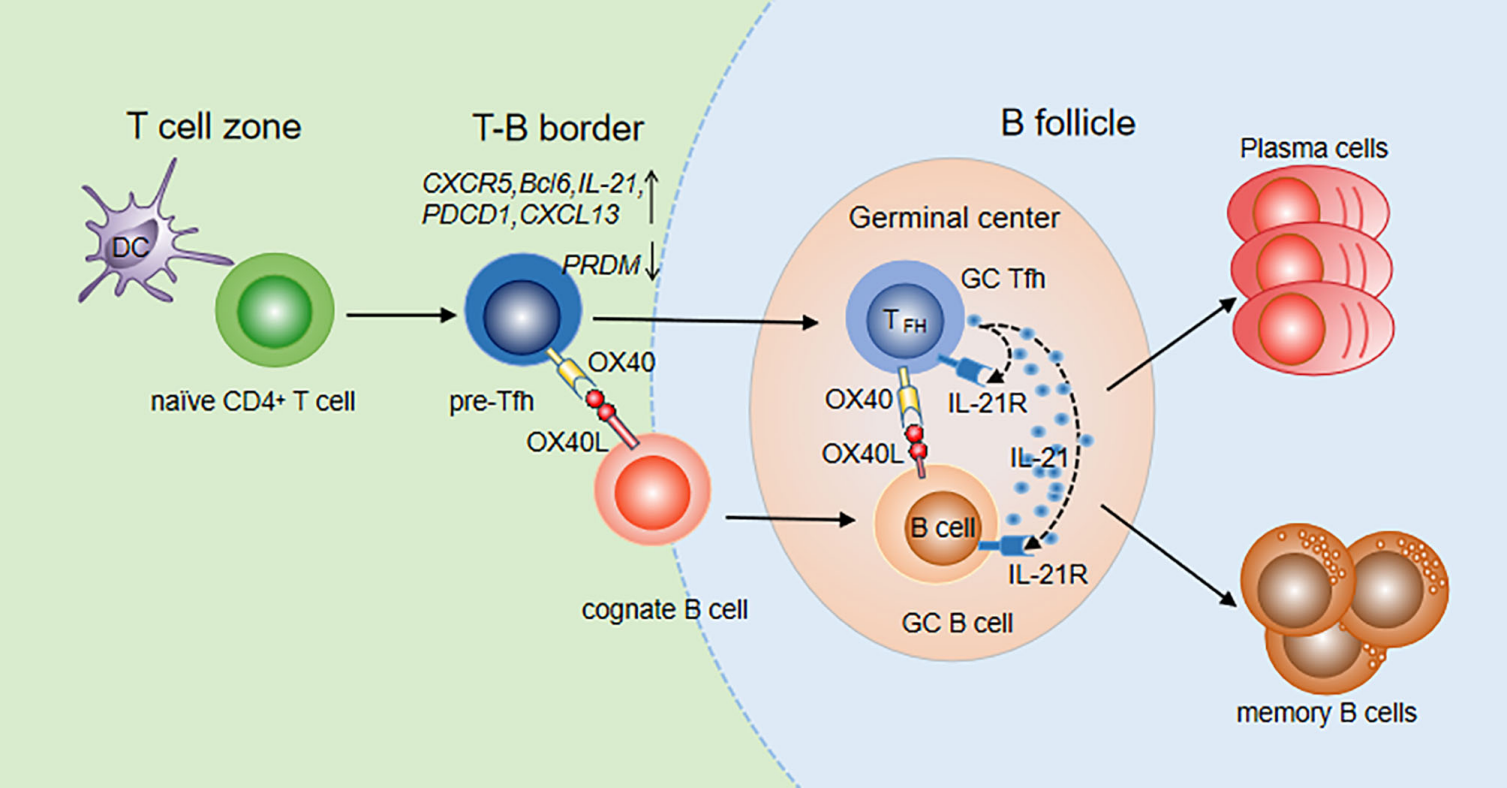
OX40/OX40L signaling in Tfh differentiation and function. Tfh differentiation occurs at the time of DC priming. Upregulation of OX40 on pre-Tfh cells promotes their accumulation at the T-B border. With the interaction of cognate B cells, OX40 signaling contributes to Tfh maintenance, maturation and migration to B follicles. Meanwhile, B cells also move to B follicles to further interact with Tfh cells. Bidirectional OX40/OX40L signaling promotes both GC Tfh and GC B cell differentiation. With the help of GC Tfh cells, B cells differentiate into plasma cells and memory cells.

CXCR5 is one of the most widely used markers to identify Tfh cells. Initially, OX40 *in vitro* was reported to induce CXCR5 mRNA transcription in activated mouse T cells, indicating that OX40 signaling may promote differentiation of Tfh cells by upregulating CXCR5 expression (71, 72). Then, the OX40/OX40L signal was found to upregulate multiple Tfh genes, including *CXCR5, Bcl-6, IL-21, CXCL13* and *PDCD1*, in both naïve and memory Th cells and to downregulate the expression of the transcription factor *PRDM1*, which inhibited the generation of Tfh cells (72). The upregulation of Bcl-6 and downregulation of PRDM1 fully demonstrated the important role of OX40 signaling in Tfh cell differentiation. Jacquemin et al. further compared the expression of Tfh genes after stimulation with OX40, IFN-γ and IL-12 which is an inducer of Tfh cells, and found that OX40 and IL-12 promoted naïve Th cells to express Tfh cell-related genes at similar levels (72). OX40 signaling is more efficient than IL-12 signaling at inducing memory Th cells to upregulate Tfh genes. The cooperation of the two signals can further increase the expression of *CXCR5* and *IL-21* on memory Th cells. In addition, it was shown that after 8-15 days of infection with vaccinia virus (VacV) in OX40-deficient mice, the numbers of Tfh and GC Tfh cells were significantly reduced compared with those in wild-type mice, indicating a critical role of OX40 in Tfh maturation (69). Prior studies reported that the interaction of OX40 and OX40L can also promote accumulation of CD4^+^ T cells in the T/B boundary and B cell follicles in mouse models with protein Ag immunization (73). Recently, Tahiliani V et al. visualized OX40L-expressing DCs and B cells at the T/B borders and in the follicle and GC, in direct association with OX40^+^ Tfh cells in these areas (69). The interaction between Tfh cells and DCs or B cells is very important for further Tfh differentiation and Tfh maintenance. Therefore, OX40/OX40L signaling promotes not only Tfh generation but also Tfh maturation and maintenance (**Figure 1**).

### Enhancement of Tfh Functions

OX40L was found to be expressed in the GCs and surrounding areas, suggesting that OX40/OX40L signaling may play a role in formation of the GC. Li Y et al. constructed a recombinant rabies virus (RABV) mouse model (LBNSE-OX40L) which overexpressed OX40L and found that Tfh cells and GC-B cells significantly increased after RABV infection (74). Deletion of OX40L in B cells in the SLE mouse model resulted in an improved disease index and a decreased number of plasma cells and GC-B cells (75). In OX40-deficient mice, GCs could not be built up even if activated B cells from wild-type mice were injected. In contrast, GCs could be formed and expanded when B cells from OX40-deficient mice were injected into wild-type mice (69), suggesting that the OX40/OX40L signal is essential for the formation of GCs. In addition, reduced Ab production has been found in animals that lack the OX40 molecule. The interaction of OX40L^+^ B cells and OX40^+^ Tfh cells has been observed in T-B border and GCs in mice infected with VacV, further indicating the roles of OX40/OX40L signaling in Tfh helping B cells. During their interaction, a bidirectional OX40/OX40L signal occurs. On the one hand, OX40 on Tfh cells can receive signals from OX40L expressed on B cells to promote secretion of cytokines such as IL-21, which further assists B cell activation and antibody production. On the other hand, B cells can receive the OX40L reverse signal from Tfh to directly expand B cell clones and promote GC formation (**Figure 1**).

### OX40-Initiated Signaling Pathways in Tfh Cells

**Figure 2:**
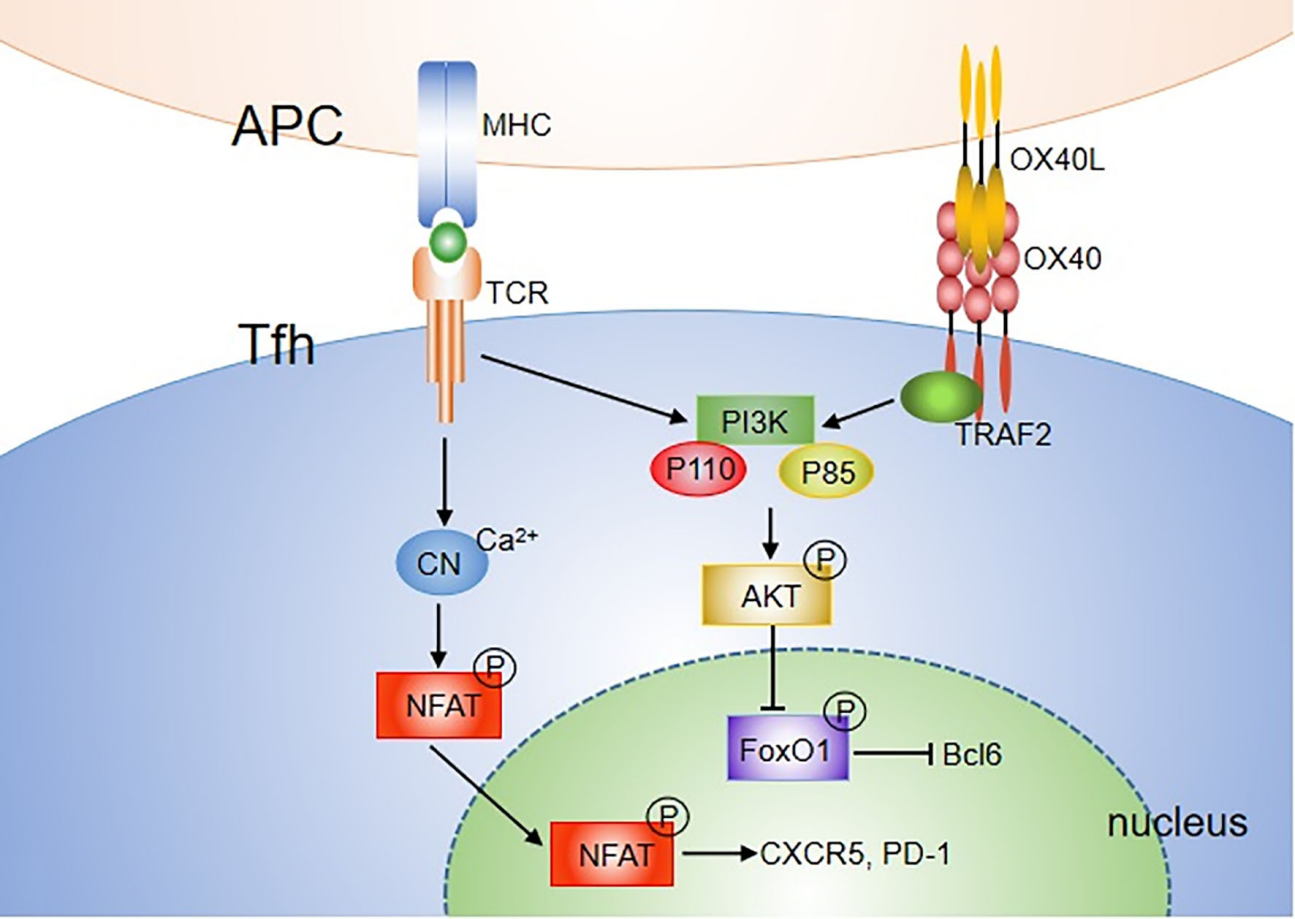
OX40 signaling pathways mediating Tfh differentiation. Both TCR and OX40 can activate PI3K, including P110 and P85 subunit, further leading to phosphorylation of AKT. pAKT then phosphorylates the FOXO1 transcription factor, which can subsequently be exported out of the nucleus and degraded. FOXO1, which represses Bcl6, is recognized as an inhibitor of Tfh differentiation. In addition, OX40 synergizes with TCR to allow Ca^2+^ influx and nuclear accumulation of NFATc1 and NFATc2. Overall, OX40 may mediate Tfh cell activity by augmenting TCR signaling *via* the NFAT or PI3K-Akt pathway.

There are two main OX40/OX40L signal transduction pathways in T cells. One is the antigen-independent NF-κB pathway, and the other is the antigen-dependent PI3K-Akt pathway. Binding of OX40L results in trimerization of OX40 monomers and recruitment of TRAF2, 3 and 5 (46, 47). TRAF2 has been characterized as an adaptor molecule that can lead to activation of NF-κB signal and recruitment of PI3K. For the NF-κB pathway, engagement of OX40 on activated/effector T cells by OX40L recruits not only the TRAF-RIP-IKKα/β/γ complex, but also the PCARMA1–BCL10–MALT1–PKCθ complex (76). This signalosome directly controls NF-κB activation without antigen/TCR engagement. The TRAF-RIP-IKKα/β/γ signaling complex mediates phosphorylation and degradation of IκBα, leading to activation of NF-κB1 and entry of p50 and RelA into the nucleus, which is sufficient to provide survival signals to T cells in the absence of antigens. The CARMA1–MALT1–BCL10–PKCθ complex forms the signalosome with OX40 in the immune synapse, which plays a major role in promoting prolonged NF-κB activity and survival of effector T cells during late-phase T cell responses when antigen is cleared. OX40 can also induce phosphorylation of IKKα and activation of NIK, which activates the noncanonical NF-κB2 pathway (46, 77, 78). For the PI3K-Akt pathway, after ligation of OX40L, OX40 was found to assemble a signaling complex that contains TRAF2, PKB and its upstream activator PI3K (79, 80). It only induced strong phosphorylation and functional activation of the PI3K-Akt pathway when Ag was presented. Thus, OX40 can augment TCR signaling *via* the PI3K-Akt pathway. In addition, OX40 synergizes with TCR to allow Ca^2+^ influx and nuclear accumulation of NFATc1 and NFATc2 (78) (**Figure 2**).

The NF-κB pathway is also involved in Tfh cell proliferation and survival (81). Blocking molecules in the NF-κB1 and NF-κB2 pathways inhibits the Bcl-6 expression on CD4^+^ T cells. However, the effect is independent of OX40 signaling (72). Strong and durable TCR signals can also contribute to promoting Th cell differentiation to the Tfh lineage and their proliferation (82–84). A recent study showed that stimulation with anti-CD3 and anti-CD28 beads promoted the expression of multiple Tfh molecules including CXCR5, IL-21, CD40L and Bcl-6, in a dose-dependent manner. The combination of OX40 and TCR signals further upregulated the expression of Tfh molecules, indicating that OX40 signaling promotes Tfh differentiation by enhancing TCR signaling. PI3K activity is an essential component of pathways driving Tfh cell and GC formation (85). Both TCR and OX40 are characterized as strong activators of both the PI3K and Akt signaling pathways. Thus, OX40 may mediate Tfh cells by augmenting TCR signaling *via* the PI3K-Akt pathway. Another way for OX40 to regulate Tfh cells may be through NFAT, which has been shown to be essential for effective Tfh development (**Figure 2**) (86).

## Implication of Tfh in Autoimmune Diseases and Immune Therapy Through OX40/OX40L Signaling

Tfh cells and OX40/OX40L have both been reported to be associated with autoimmune diseases both in humans and mice (**Table 2**). Since many autoimmune diseases such as SLE, RA and Graves’ diseases are autoantibody-mediated, the critical roles of Tfh cells in these diseases are obvious (87–101). Tfh cells enhance the intensity and duration of the GC response and promote autoantibody production. In lupus nephritis lesions, Tfh-like cells expressing PD-1, ICOS, IL-21 and Bcl-6 were observed to form ectopic GCs. In addition, an increased population of circulating Tfh cells was identified in SLE patients (102). In salivary gland tissues and peripheral blood of patients with Sjogren’s syndrome (SS), the numbers of CD4^+^CXCR5^+^ Tfh cells were significantly increased along with abnormal B cells and plasma cells, suggesting that Tfh cells participate in the pathogenesis of SS by promoting B cell maturation (103). Moreover, multiple studies have demonstrated that OX40 is expressed on pathogenic T cells in autoimmune disease (42, 104–112). Farres et al. found that compared with healthy people, CD4^+^ T cells in SLE patients express high levels of OX40, and the disease activity index is positively correlated with the number of CD4^+^ T cells expressing OX40. The disease is improved after treatment with an anti-OX40L monoclonal antibody (113, 114). Yoshioka et al. found that T lymphocytes in synovial fluid and synovial tissue of RA patients express OX40, and secondary lymphocytes in synovial tissue express OX40L, suggesting that the OX40/OX40L interaction may play a key role in RA occurrence and development (34). Graves’ disease (GD) is an autoimmune thyroid disease, with clinical manifestations that primarily include ophthalmopathy, goiter and hypermetabolic syndrome (115, 116). We have found that OX40/OX40L was abnormally and persistently coexpressed on CD4^+^ T cells from GD patients, and the coexpression level was closely related to TRAb (117).

**Table 2:** OX40, OX40L expression and Tfh cells in autoimmune diseases.

Disease	OX40	OX40L	Tfh
Systemic lupus erythematosus (SLE)	Upregulated OX40 expression on peripheral T cells (108)	Upregulated OX40L expression on myeloid APCs (72)	Increased Tfh in patients with active SLE (108)
Rheumatoid arthritis (RA)	Upregulated OX40 expression on T cells in synovial fluid and blood (34, 67, 119) Upregulated OX40 expression on circulating CD4^+^CD28^-^ T cells (116) Upregulated OX40 expression on circulating Tfh17 cells (67)	Upregulated OX40L expression on sublining cells in synovial tissue (34) and on monocytes and B cells in blood (90)	Increased circulating Tfh cells and Tfh17 cells (118)
Type 1 Diabetes (T1D)	Increased circulating CD4^+^CD25^high^ OX40^+^ T cells in children with newly diagnosed T1D (94)		Increased circulating Tfh cells in newly diagnosed T1D children (102) Increased circulating Tfh cells in T1D patients (88, 89)
Graves’ diseases	Upregulation of OX40 on circulating CD4^+^ T cells (117)	Upregulation of OX40L on circulating CD4^+^ T cells (117)	Increased circulating Tfh and Tfh2 cells (90) Elevated Tfh cells in thyroid tissues (77, 78)
Multiple sclerosis	Downregulation of OX40 expression on circulating CD4^+^ T cells after treatment with natalizumab (95) The presence of OX40^+^ T cells in brain tissue (106)		Increased circulating IL-21-producing Tfh-like cells (96) Increased Tfh/Tfr ratio associates with abnormal IgG production in blood and CSF (92)
Myasthenia gravis	Upregulation of OX40 expression on circulating CD4^+^ T cells (107) and thymic CD4^+^ T cells adjacent to GC (108).	The presence of OX40L^+^ mononuclear cells in thymic GC (108).	Increased circulating Tfh cells (102) Increased circulating Tfh17 cells in MuSK-antibody positive patients (93).
Sjogren syndrome	Upregulation of OX40 expression on circulating CD4+ T cells (108)	Upregulation of OX40L expression on circulating B cells and monocytes (108)	Increased circulating Tfh and Tfh17 cells (94) Localization of Tfh cells in salivary glands (95)
lupus mouse	Upregulation of OX40 expression on CD4^+^ T cells in the spleen and kidney of NZB/WF1 mouse (109)		Expanded Tfh cells in spleen of MRL/lpr mouse (96) Tfh cells infiltrating the brain of murine neuropsychiatric lupus in MRL/lpr mouse (97)
Collagen-induced arthritis mouse	Upregulation of OX40 expression on CD4^+^ T cells in joints (110) and spleen (111) Upregulation of OX40 expression on CD4^+^CD28^-^ T cells in spleen (118)	Upregulation of OX40L expression on APCs in spleen (98)	Increased Tfh cells in the spleen (111)
T1D mouse	Upregulation of OX40 expression on CD4^+^ and CD8^+^ T cells in pancreatic lymph nodes and spleen of NOD mouse prior to diabetes onset (120)	Upregulation of OX40L expression on dendritic cells in pancreatic lymph nodes late during NOD development (120)	Increased Tfh cells in the pancreatic lymph node and the pancreas of DO11×RIP-mOVA mouse (99)
Autoimmune encephalomyelitis (EAE) model	Upregulation of OX40 expression on CD4+ T cells in spleen and brain tissue (42, 106) OX40 expression selectively on autoantigenic CD4^+^ T cells from the inflammatory site in spinal cord or brain (111, 112)	Upregulation of OX40L expression on CD11b^+^ cells and vascular endothelial cells in central nerous system (106, 121)	Increased Tfh cells in ectopic lymphoid structures in spinal cords (100)

Recent data for SLE and RA showed that high OX40 and OX40L expression may be involved in the pathogenesis of autoimmune diseases by enhancing Tfh functions. Jacquemin et al. also reported that OX40L^+^ myeloid cells are visualized in skin and kidney tissues from SLE patients. OX40 engagement upregulated the expression of several Tfh-associated molecules in T cells from lupus patients, including Bcl6, CXCR5 and IL-21, showing that Th cells in an OX40L-rich environment may receive OX40 signaling to promote Tfh development. The percentage of OX40L^+^ myeloid cells in blood was significantly higher in active patients than in inactive patients and positively correlated with peripheral Tfh cell frequencies, indicating that the Tfh response was enhanced by the OX40 signal. Moreover, myeloid cells expressing OX40L can also impair Treg and Tfr functions by suppressing Tfh-dependent B cell activation and immunoglobulin secretion in SLE. OX40-overexpressing Tfh cells, especially Tfh 17 cells, were found to be increased in RA and a murine model. In vitro coculture experiments showed enhanced hyposialylation by the Tfh cells *via* OX40. Blockade of OX40 signaling prevented arthritis development by reducing Tfh17 cells and recovering autoantibody salivation (67). Therefore, upregulated OX40 signaling plays a crucial role in the development of autoimmune diseases by enhancing Tfh functions directly or indirectly. Thus, targeting OX40/OX40L signaling may be an effective strategy for these diseases.

OX40/OX40L blockade *in vivo* is generally effective in many models with autoimmune diseases, mainly by inhibiting activation and migration of CD4^+^ T cells and altering cytokine production (34, 118, 119) (**Table 3**). When a blocking anti-OX40L antibody was given to NOD mice at 12 weeks of age, the incidence of diabetes was reduced (120). In EAE mice, anti-OX40L antibody blockade leaded to decline of clinical score and reduction of spinal cord T cell infiltration (121, 122). There are also some clinical trials targeting OX40/OX40L in development. OX40L-blocking antibodies were reported to ameliorate antigen-driven Th2 responses in mouse and nonhuman primate models of asthma (123). An anti-OX40 antibody, GBR 830 in phase II study showed significant clinical improvement in patients with moderate to severe atopic dermatitis (125) (**Table 3**). Combined OX40L and mTOR blockade in nonhuman primate graft-versus-host disease (GVHD) model prolonged survival by controlling effector T cell activation while preserving Treg reconstitution (126). However, the treatment with a humanized anti-OX40L mAb has no effect on allergen-induced airway responses in mild asthmatic patients (124). Timing and dosing of clinical intervention may be critical for the efficacy.

**Table 3:** Therapeutic effects of OX40/OX40L blockade *in vivo*.

Disease	Model	Intervention and effect
Rheumatoid arthritis (RA)	Collagen-induced arthritis (CIA) mouse	Anti-OX40L mAb ameliorated clinical score and suppress IFN-*γ* and anti-CII Ig2a production (34)
	CIA mouse	Anti-OX40L mAb reduced the proinflammatory responses and ameliorated arthritis development (118)
	CIA mouse	Treatment with the anti-OX40 Fab′PEG blocking antibody and the OX40L:Ig fusion protein delayed the time of onset of arthritis and reduced the overall clinical score (119)
Type 1 Diabetes (T1D)	NOD mouse	Anti-OX40L mAb given to NOD mice at 12 weeks of age prevented diabetes development (120)
Multiple Sclerosis	EAE mouse	Anti-OX40L antibody leaded to decline of clinical score and reduction of spinal cord T cell infiltration (121, 122)
Asthma	mouse and nonhuman primate models	Anti-OX40L mAb inhibited Th2 lung inflammation (123)
	Mild atopic asthmatic patients	A humanized anti-OX40L mAb has no effect on allergen-induced airway responses despite partial and transient reduction in total IgE and airway eosinophils (124)
Atopic dermatitis	Patients with moderate to severe syndrome	Blocking anti-OX40 antibody showed significant clinical improvement (125)
Graft-versus-host disease (GVHD)	nonhuman primate model	Combined OX40L and mTOR blockade prolonged survival by controlling effector T cell activation (126)

## Conclusion

Providing help for B cell development and GC reactions is the most crucial function of Tfh cells, which lead to high-affinity antibody production. Thus, increased activity of Tfh cells plays a pathogenic role in a wide range of autoimmune diseases, in both mice and humans. The differentiation of Tfh cells requires not only TCR signaling, cytokines and antigen stimulation but also costimulatory signals, such as ICOS/ICOSL and OX40/OX40L. As an important marker of Tfh cells, OX40 can promote Tfh generation and contribute to maintenance of Tfh and GC B cells at later times. OX40 synergizes with ICOS to maximize and prolong the Tfh response. Therefore, upregulation of OX40 and OX40L may induce abnormal activation of Tfh cells and excessive production of autoantibodies, leading to the development of autoimmune diseases.

Given that blocking OX40/OX40L signaling has shown great therapeutic effects in some mouse models of autoimmune diseases, targeting OX40/OX40L is promising as a new therapeutic approach for these diseases. However, the efficacy data of clinical trials are currently limited. Further studies are needed for clinical intervention since many factors, such as dose and time point, influence the effect. Moreover, controversial results have been obtained regarding the roles of the OX40/OX40L axis in regulation of Tfh responses. Whether other factors may impact the roles of OX40L in Tfh cells needs also to be further investigated.

## Author Contributions

QW and ZS organized and supervised the entire manuscript. NF contributed to the sections on Tfh cell differentiation and functions and the OX40 and OX40L molecules. XF contributed to the sections on OX40 signaling in Tfh cells and the implication of Tfh cells in autoimmune diseases. All authors contributed to the article and approved the submitted version.

## Funding

This work was supported by grants from the National Natural Science Foundation of China (No. 81373184), the Key University Science Research Project of Jiangsu Province (20KJA180002), and the Center of Engineering Technology R&D of the Department of Jiangsu Province Education (No. 201826).

## Conflict of Interest

The authors declare that the research was conducted in the absence of any commercial or financial relationships that could be construed as a potential conflict of interest.
